# A predictive coding perspective on autism spectrum disorders

**DOI:** 10.3389/fpsyg.2013.00019

**Published:** 2013-01-28

**Authors:** Jeroen J. A. van Boxtel, Hongjing Lu

**Affiliations:** ^1^Department of Psychology, University of CaliforniaLos Angeles, CA, USA; ^2^Department of Statistics, University of CaliforniaLos Angeles, CA, USA

**A commentary on**

**When the world becomes ‘too real’: a Bayesian explanation of autistic perception**

by Pellicano, E., and Burr, D. (2012). Trends Cogn. Sci. 16, 504–510.

**Figure 1:**
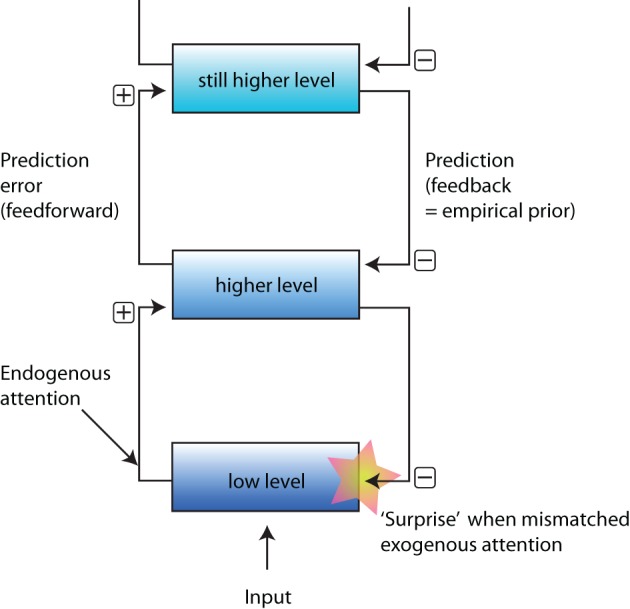
**A schematic representation of the predictive coding framework.** Input arrives from the sensory organs, and is processed in a “low-level” area. This processed information is sent to a higher area. Based on this input the higher area tries to explain, and predict the sensory data, and feeds back a prediction. The prediction is more or less equivalent to the “prior” in the Bayesian framework. The prediction is subtracted from the activity at the lower level, and the residual is the “prediction error.” The prediction error may be equivalent to “exogenous attention.” Finally, endogenous attention may influence the feedforward information.

In a recent article entitled “When the world becomes ‘too real’: Bayesian explanation of autistic perception,” Elizabeth Pellicano and David Burr (Pellicano and Burr, 2012b) introduce an intriguing new hypothesis, a Bayesian account, concerning the possible origins of perceptual deficits in Autism Spectrum Disorder (ASD). This Bayesian account explains why ASD impacts perception in systematic ways, but it does not clearly explain how. Most prominently, the Bayesian account lacks connections to the neural computation performed by the brain, and does not provide mechanistic explanations for ASD (Rust and Stocker, 2010; Colombo and Series, 2012). Nor does the Bayesian account explain what the biological origin is of the “prior”—the essential addition of the Bayesian models. In Marr's terminology (Marr, 1982), Pellicano and Burr paper proposes a computational-level explanation for ASD, but not an account for the other two levels, representation and implementation. We propose that a predictive coding framework (schematized in Figure 1) may fill the gap and generate a testable framework open to further experimental investigations.

In Pellicano and Burr's general Bayesian approach, perception is based on the integration of stimulus information (encapsulated in the likelihood) and regularizing (contextual) information based on previous experience (the “prior”). Often, the prior draws perception away from the veridical stimulus characteristics [e.g., people perceive a Kanizsa triangle above three circles, instead of three pac-men: see Figure 1 in Pellicano and Burr (2012b)]. Pellicano and Burr suggest that people with ASD have weak priors compared to the typically-developing population, explaining a key finding that autistic observers are less influenced by contextual information, and hence see the world more accurately (as it actually is), as their perception is less modulated by experience. This Bayesian account provides an explanation for the bias favoring local over global processing in ASD.

The predictive coding framework provides a natural implementation of the prior used in the Bayesian model proposed by Pellicano and Burr. In predictive coding schemes, higher brain areas attempt to “explain” input from lower brain areas, and then project these predictions down to lower areas, where the predicted sensory information is subtracted from the input (i.e., predicted information is discounted). This feedback operates in a hierarchical manner (Figure 1), and the predictions fed-back to lower areas constitute the (empirically-derived) “priors” (Feldman and Friston, 2010). Such empirical priors have been computationally implemented (Rao and Ballard, 1999; Feldman and Friston, 2010), and thus are open to experimental scrutiny. An added advantage of this framework is that it naturally explains the often-observed decrease in global processing in people with ASD, and concomitant increase in local processing (Happé and Frith, 2006; Mottron et al., 2006).

The predictive coding framework also provides an elegant way to implement both endogenous (top-down) and exogenous (bottom-up) attention within the same framework. The framework can therefore guide detailed investigations of whether perceptual deficits in ASD are due to malfunctioning of certain higher-level brain areas, or instead due to an attentional bias toward lower-level stimulus characteristics (Plaisted, 2001; Mottron et al., 2006).

Exogenous attention is linked to the prediction error in the predictive coding framework. Specifically, when the predictions (“priors”) do not match the input, expectations are violated, and a prediction error (i.e., the difference between the expected and the observed sensory information) is generated at lower levels. The prediction error constitutes a “surprise” (Feldman and Friston, 2010), which can be thought of as a trigger for exogenous attention. With decreased high-level processing in ASD (e.g., Brosnan et al., 2004; Happé and Frith, 2006), predictions are presumably less precise (or less strong, i.e., hypo-priors; Pellicano and Burr, 2012b), and thus prediction errors (“surprises”) will increase. As a result, the sensory systems of people with ASD will be constantly bombarded by new “surprises”, and hence overloaded with sensory stimulation.

Endogenous attention can also be readily included in the predictive coding framework as a modulation of feedforward information (as explained in Feldman and Friston, 2010). Empirical evidence for such modulation exists (Zhang and Luck, 2009). Within the predictive coding framework, decreased influence of higher visual areas on perception, manifested in decreased activity (e.g., Belmonte et al., 2004; Schultz, 2005) or decreased (functional) connectivity (Just et al., 2004; Liu et al., 2011), could be due to decreased functioning of higher levels, or alternatively to decreased endogenous modulation of attention (Mottron et al., 2006), or both. Developing quantitative computational models may help us disentangle these possibilities.

The predictive coding framework may also provide valuable insights into the developmental origins of ASD. Because of the recurrent nature of the predictive coding framework, it is possible that a dysfunction in one level causes a dysfunction in another level, which in turn feeds back to create a vicious circle. If this cycle occurs during development, it could potentially spiral out of control, contributing to ASD. Such scenarios go beyond a simple Bayesian account based on priors and likelihoods and could be investigated with computational models in the future (Rao and Ballard, 1999; Feldman and Friston, 2010).

Finally, in a recent comment on Pellicano and Burr's paper, Brock (Brock, 2012) suggested that instead of hypo-priors, one may assume that people with ASD have reduced sensory noise. Although this is theoretically possible, Pellicano and Burr countered (Pellicano and Burr, 2012a) that there is in fact experimental evidence for increased neural noise in ASD. We would add that the hypothesis of reduced sensory noise also predicts a reduced variance in the intra-individual perceptual responses to identical (visual) stimuli, whereas a hypo-prior would be associated with an increase in variance. Although the literature on this issue is not extensive, intra-individual response time variability is reportedly greater in ASD than in the typical population (Geurts et al., 2008).

In summary, the predictive coding framework complements the Bayesian approach introduced by Pellicano and Burr, providing a general account of why certain perceptual, and potentially social deficits (cf., Kilner et al., 2007) exist, and how biological substrates and computational mechanisms can give rise to these deficits in ASD.
